# Comparative efficacy of various exercise interventions on sleep in patients with cognitive impairment: a systematic review and meta-analysis

**DOI:** 10.3389/fneur.2024.1300459

**Published:** 2024-02-01

**Authors:** Junlei Zhang, Yan Liu, Qingling Sun, Jing Shi, Jingnian Ni, Ting Li, Ziyi Long, Mingqing Wei, Jinzhou Tian

**Affiliations:** ^1^Dongzhimen Hospital, Beijing University of Chinese Medicine, Beijing, China; ^2^Neurology Center, Dongzhimen Hospital, Beijing University of Chinese Medicine, Beijing, China

**Keywords:** exercise, sleep quality, cognitive impairment, Alzheimer’s disease, randomized controlled trials, systematic review and meta-analysis

## Abstract

**Background:**

Sleep disturbances are an early indicator of cognitive impairment and exacerbate its progression. While pharmacological treatments for sleep disorders exist, their side-effect profile includes an increased risk of falls and the potential to exacerbate cognitive impairment. Non-pharmacological treatments such as physical exercise should be considered. However, uncertainties persist. We aimed to assess the potential benefits of exercise interventions on sleep in patients with cognitive impairment and determine the specific effects of various exercise modalities.

**Materials and methods:**

A systematic search was performed on seven databases for eligible studies published before Nov 2022. Randomized controlled trials of exercise for patients with cognitive impairment (mild cognitive impairment and Alzheimer’s disease) were included. All analyses were conducted using RevMan version 5.4. Meta-analysis and The Grading of Recommendations Assessment Development and Evaluations (GRADE) quality ratings were performed on sleep quality and objective sleep data.

**Results:**

A total of 8 randomized controlled trials were included with a sample size of 486 subjects. For patients with cognitive impairment, physical exercise had a beneficial effect on sleep quality [MD = −3.55 (−5.57, −1.32), *Z* = 3.13, *p* = 0.002] and total sleep time [MD = 33.77 (23.92, 43.62), Z = 6.72, *P* < 0.00001]. No improvement was found in sleep efficiency and nocturnal awakening time. Subgroup analysis showed that multi-component exercise produced superior results.

**Conclusion:**

Physical exercise may improve sleep quality and total sleep time for patients with cognitive impairment. Multi-component exercise designed individually is more effective. Large-scale randomized controlled trials with objective sleep outcome measurements are warranted.

**Clinical trial registration**: https://www.crd.york.ac.uk/prospero/, identifier: CRD42022377221.

## 1 Introduction

Dementia constitutes a formidable challenge to global public health, primarily contributing to disability and mortality (1). As estimations by the World Health Organization reported, in 2021, over 55 million individuals are projected to suffer from this debilitating ailment, and this figure is predicted to soar to 150 million by 2050 (2). In light of the overwhelming burden that dementia poses, there is an urgent need to devise effective preventive and therapeutic interventions that can impede the progression of this malady and thereby alleviate its socioeconomic impact.

Approximately 45% of individuals with Alzheimer’s disease (AD) reportedly exhibit symptoms of sleep disorders (3, 4). These complaints are not merely indicative of the initial stages of AD (5); they may also serve as a potential risk factor for and exacerbate the pathological progression of AD (6). The bidirectional relationship between AD and sleep disruption has been corroborated by mounting evidence regarding their respective pathophysiology (7–9). Hence, early intervention in the sleep patterns of cognitively-impaired patients has emerged as a crucial preventative and therapeutic approach for AD.

Benzodiazepines, a class of drugs commonly used to treat anxiety and sleep disorders, have been previously linked to an increased risk of AD with prolonged use (10). Additionally, patients with AD often exhibit abnormal behaviors associated with dementia, for which atypical antipsychotic medications have been frequently prescribed to treat sleep problems. However, a study has reported that such medications were associated with a decline in cognitive function, as measured by the Mini-mental State Examination (−2.4 points) and Alzheimer’s Disease Assessment Scale-cog (+4.4 points), over a 36-week treatment period, which was consistent with 1 year’s deterioration compared to placebo (11). The well-documented side effects of sedative-hypnotic drugs further emphasize the need for caution when prescribing them to individuals with neurodegenerative diseases. In light of this, the National Institute for Health and Care Excellence recommends non-pharmacological treatments as a first-line option for sleep problems in individuals with dementia, with physical activity being a potential option (12). The effectiveness of physical exercise in improving sleep quality involves intricate physiological processes that encompass several systems, including the central nervous system, endocrine system, metabolism, and body temperature regulation (13). However, the optimal form, intensity, duration, and amount of exercise for short- or long-term effectiveness vary, with gender potentially influencing the results (14).

The impact of physical exercise on cognitive function and functional independence in patients with cognitive impairment has been extensively studied in the past (15, 16). However, scant attention has been paid to the influence of physical activity on sleep patterns. In this systematic review and meta-analysis, we sought to comprehensively explore the effects of physical activity on sleep problems in individuals with cognitive impairment. Our goal was to identify the most efficacious exercise therapies to address this population’s sleep disorders and provide a valuable reference for clinicians and caregivers.

## 2 Materials and methods

This study presented a systematic review and meta-analysis (SR-MA) conducted in strict adherence to the guidelines set forth by the Preferred Reporting Items for Systematic Reviews and Meta-Analyses (PRISMA) 2020 (17). The SR-MA protocol was duly registered with the Prospective Register of Systematic Reviews (PROSPERO: CRD42022377221), thus ensuring the transparency and methodological rigor of our investigation.

### 2.1 Information sources and search strategy

Five international databases, namely Web of Science, PubMed, Embase, Cochrane Central Register of Controlled Trials, and Scopus, along with two Chinese databases, namely Wanfang Data and China National Knowledge Infrastructure, were searched online up until November 2022. Additionally, eligible trials were identified through the World Health Organization International Clinical Trials Registry Platform and ClinicalTrials.gov. The reference lists of relevant articles were manually searched for additional studies on the program to supplement the search. The keywords used in the search included AD, dementia, mild cognitive impairment, cognitive function, sleep, and exercise. A detailed search strategy was employed, and the PubMed search strategy is presented in Table 1.

**Table 1:** Search strategy in PubMed.

Step	Search strategy
#1	Search ((Alzheimer disease[MeSH Terms]) OR (Cognitive Dysfunction[MeSH Terms])) OR (Dementia[MeSH Terms])
#2	Search (exercise[MeSH Terms]) OR ((((((((((((((((exercises[Title/Abstract]) OR physical activit*[Title/Abstract]) OR training*[Title/Abstract]) OR danc*[Title/Abstract]) OR yoga[Title/Abstract]) OR taichi[Title/Abstract]) OR wuqinxi[Title/Abstract]) OR baduanjin[Title/Abstract]) OR yijinjing[Title/Abstract])) OR (aerobic[Title/Abstract])) OR (weight lifting[Title/Abstract])) OR (resistance[Title/Abstract])) OR (motor activity[Title/Abstract])) OR (strength[Title/Abstract])) OR (structured limbs-exercise program[Title/Abstract])) OR (resistance exercise[Title/Abstract])
#3	Search (((((((randomized controlled trial[Title/Abstract]) OR (controlled clinical trial[Title/Abstract])) OR (randomized[Title/Abstract])) OR (placebo[Title/Abstract])) OR (clinical trials[MeSH Terms])) OR (randomly[Title/Abstract])) OR (trial[Title/Abstract])) NOT (animals[Title/Abstract])
#4	((((((((((((((sleep[MeSH Terms]) OR (sleep disorder[MeSH Terms])) OR(latency persistent sleep[Title/Abstract])) OR (rapid eyemovement sleep[Title/Abstract])) OR (number of awakenings[Title/Abstract])) OR (number of arousals[Title/Abstract])) OR (non-rapid eye movement sleep stages[Title/Abstract])) OR (sleep efficiency[Title/Abstract])) OR (rapid eye movement sleep[Title/Abstract])) OR (total sleep time[Title/Abstract])) OR (wake after persistent sleep onset[Title/Abstract])) OR (Polysomnography[Title/Abstract])) OR (Actigraph[Title/Abstract])) OR (Sleep quality [Title/Abstract])) OR (circadian rhythm[Title/Abstract])
#5	#1 AND #2 AND #3 AND #4

### 2.2 Eligibility criteria and selection process

Two independent reviewers screened all studies (Zhang J, Wei M). Duplications were eliminated using the software of EndNote 20. They screened out all potentially relevant studies by reading the titles and abstracts which met the eligibility criteria. Disagreements were resolved in consultation with a third expert (Shi J) if necessary. The studies included that met the following inclusion criteria:

- Population: The participants were required to have a diagnosis of mild cognitive impairment (MCI) or dementia, and the study contained more than four adult participants (i.e., mean age ≥ 18 years).
- Intervention: The interventions included any exercise.
- Comparator: The control group must be a placebo. The comparator did not receive the intervention, sham exercise training, or other forms of exercise.
- Outcome: The studies had to report one of the following outcomes, which included self-reported or objective measured such as sleep quality, total sleep time, sleep onset latency, time to awakening after sleep onset, the number of awakenings after sleep ends, sleep maintenance time, sleep efficiency.
- Study type: The study’s design had to be a randomized controlled trial (RCT), including cluster and multi-arm RCT. Full text written in Chinese or English.
- The study was excluded for the following reasons:
- The studies of unconventional sleep protocols, such as day-night shift workers or studies of insomnia caused by experiments, conference papers, and abstracts without full text.
- The studies for which complete data were unavailable will not be included in this meta-analysis, and only narrative synthesis will be performed.

### 2.3 Data collection process and data items

Two reviewers independently extracted data using self-designed statistical forms according to the Cochrane Handbook (18) (Zhang J, Wei M), with disagreements resolved by discussion or arbitration by a third expert (Shi J). The forms included the following:

- Study characteristics (author, year of publication, title, location, and study design).
- Participant characteristics (type of cognitive impairment, sample size, cognitive function, mean age, sex ratio, and the presence or absence of sleep complaints).
- Exercise characteristics (type of exercise, duration of exercise, frequency, length of intervention, intensity, and control information).
- Outcomes (type of assessment, sleep quality, pre- and post-intervention results).

In order to discern the effects of interventions across multiple exercise modalities and investigate the determinants of sleep variation among distinct exercises, we have undertaken a process of standardization and categorization. This has entailed the establishment of specific categories for frequency, intensity, and duration per exercise, as well as the length of intervention, all under the Physical Activity Guidelines for Americans (19) and prior systematic reviews (15, 20). The details include the following:

- Type of exercise[Aerobic Exercise (AE): aiming to improve cardiovascular fitness including walking, running, or cycling; Resistance Exercise (RE): with the intent of increasing muscular strength and power using elastic bands, weight-machines; Mind–Body Exercise (MBE): aiming to improve participants’ mind–body coordination and awareness by practicing a series of controlled movements and focusing on interactions among the brain, body, mind, and behavior, such as Tachi, Baduanjin, yoga, and dance; Multi-component Exercise (ME): the combination of at least two types of exercise such as AE, RE, MBE.]
- Frequency (Low:1–2 times/week, Moderate:3–4 times/week, High:≥5 times/week)
- Duration per session (Short:<30 min, Moderate: 30–59 min, Long:≥60 min)
- Length of intervention (Short:<13 weeks/3 months, Moderate:14–25 weeks/3 months-6 months, Long:≥60 weeks/6 months)
- Intensity (Low:<60% Maximum heart rate (HRmax), Moderate:60–85% HRmax, High:>85% HRmax)

The investigation centered on evaluating sleep quality as the primary outcome. The Pittsburgh Sleep Quality Index (PSQI) was employed to measure this parameter, comprising seven scoring items with a cumulative score of 21 points. A lower PSQI score indicates superior sleep quality (21). The instrument exhibits good psychometric properties, internal consistency reliability, and structural validity and is widely employed in the comprehensive evaluation of sleep disruptions in individuals with cognitive impairment (22). As secondary outcomes, objective sleeping data were gathered through Actigraphy or polysomnography monitoring, including total sleep time, sleep efficiency, time of night awakenings, number of night awakenings, and sleep cycles.

### 2.4 Study risk of bias assessment

In accordance with the guidelines outlined in the Cochrane Handbook for Systematic Reviews of Interventions (18), two reviewers (Zhang J, Wei M) conducted an independent assessment of the eligible studies to evaluate the risk of bias for each study. In cases of disagreement between the reviewers, a third expert (Shi J) was consulted to resolve the matter. Version 2 of the Cochrane risk of bias tool for randomized trials was used to assess and categorize each included study for risk of bias as ‘high risk,’ ‘some concerns’, or ‘low risk.’ The rigorous evaluation of these domains enabled a comprehensive assessment of the risk of bias for each study, providing a thorough understanding of the quality of the evidence presented in this study.

### 2.5 Data analysis

In this meta-analysis investigation, RevMan version 5.4 was employed for data analysis. Solely continuous data were incorporated into the study, and mean differences (MD) and standard deviations (SD) were used as the primary analytical metrics. In the event of incomplete reporting of pertinent statistics, authors were contacted through electronic mail to request detailed data. In instances where the authors did not respond, we resorted to estimating the mean and standard deviation in accordance with the Cochrane Handbook, drawing upon information from the sample size, median, range, and value of *p* (18). *I*^2^ was adopted to indicate heterogeneity in this study. The degree of heterogeneity using *I*^2^ overlapping intervals was interpreted as 0–40% (might not be important), 30–60% (may represent moderate heterogeneity), 50–90% (may represent substantial heterogeneity), and 75–100% (considerable heterogeneity) (18). Suppose significant heterogeneity (more than 50 percent) is detected, the random-effects model was applicated in this meta-analysis, and, where appropriate, subgroup or sensitivity analyses are performed to explore sources of heterogeneity (18, 23). Sensitivity analyses were conducted the missed item method to assess the potential impact of individual trial results on the overall results of the meta-analysis. We did not assess publication bias for specific outcomes if the number of included RCTs was less than 10. For all analyses, the significance level is set to a probability value less than 0.05.

### 2.6 Subgroup analysis

Grouping of this meta-analysis was conducted based on geographical regions, cognitive impairment type, sleep complaints, exercise type, exercise duration, frequency, and intensity. The effects of interventions were evaluated within each subgroup and compared across subgroups to identify factors that modify intervention outcomes.

### 2.7 Certainty assessment

GRADE approach (24) was used to assess the certainty of the evidence for all domains at risk of bias in all outcomes. It was finished using the software GRADE profiler 3.6. According to the GRADE approach, the certainty of the evidence was graded as ‘very low,’ ‘low,’ ‘moderate,’ or ‘high.’

## 3 Results

**Figure 1:**
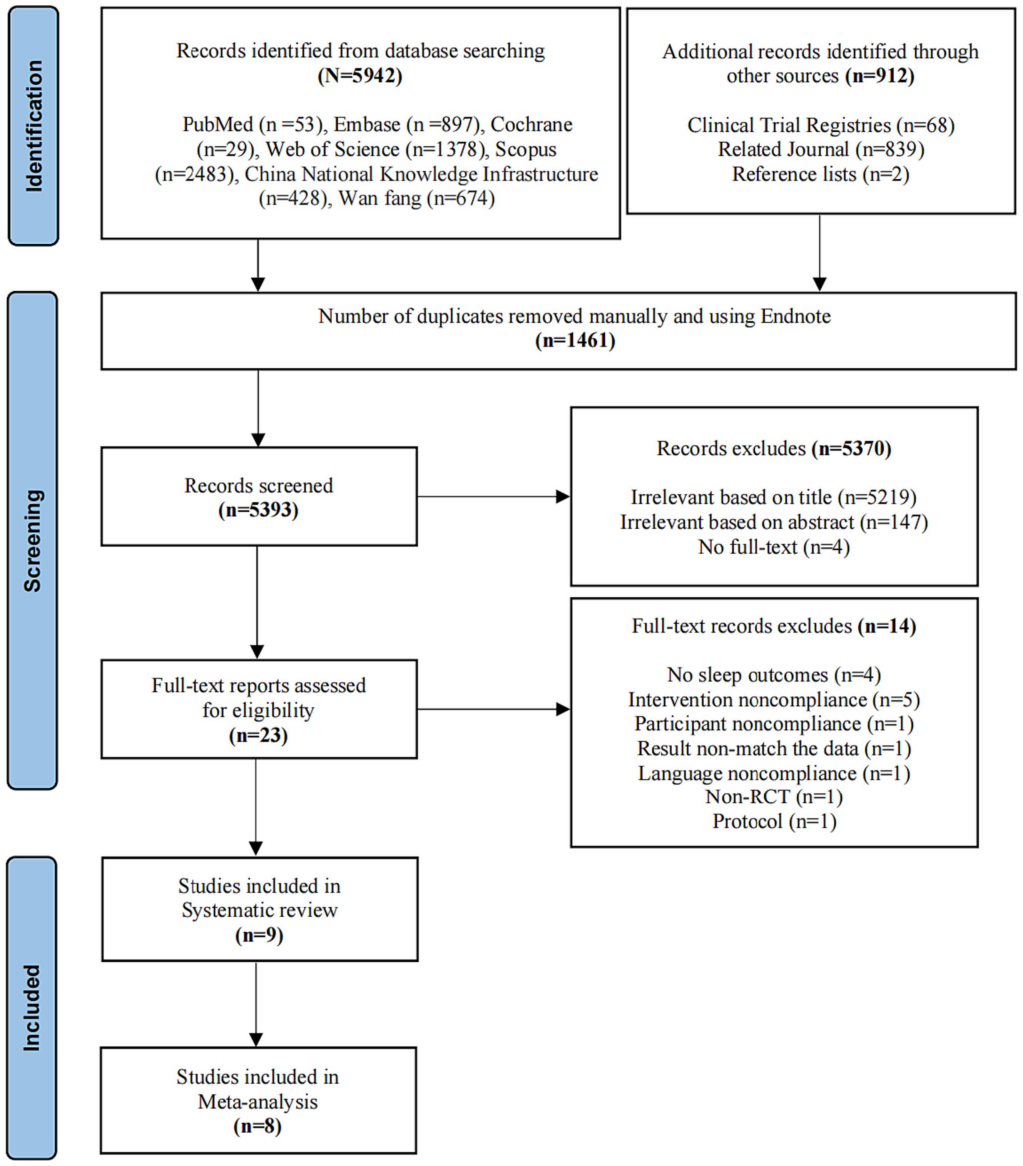
Study flow diagram.

A total of 5,942 records were retrieved from seven databases, and an additional 912 records were identified from clinical trial registries, related journals, and reference lists. The 1,461 duplicates were removed using EndNote 20 software. Subsequently, 5,370 records were excluded based on title, abstract, and full-text screenings by two independent reviewers. Among the excluded records, 14 articles were rejected for reasons such as no sleep outcome (*n* = 4), intervention noncompliance (*n* = 5), participant noncompliance (*n* = 1), language noncompliance (*n* = 1), result non-match the data (*n* = 1), non-randomized controlled trial (*n* = 1), and protocol (*n* = 1). Eventually, nine randomized controlled studies (25–33) were included in the SR-MA, with eight eligible for meta-analyses. The selection process, presented using the PRISMA flowchart, is depicted in Figure 1.

### 3.1 Study characteristics

This study presents a comprehensive analysis of nine RCTs conducted between 2011 and 2022. These trials were comprised of a two-arm RCT (29), a five-arm RCT (25), a cluster RCT (30), and six parallel-group RCTs. The majority of the trials (seven out of nine) were conducted in China, while one trial was conducted in Turkey (26) and another in the United States (33). Further information on the included studies can be found in Table 2.

**Table 2:** Characteristics of enrolled studies.

Study characteristics			Participant characteristics						Exercise characteristics					Outcome measurement	Results Mean (SD)	
Author, year	Location	Study design	Dementia/ MCI	Cognitive function Mean (SD)	Sample size	Age Mean (SD)	%Female	Identified as having sleep complaints? (Yes/No)	Type of Exercise	Duration	Frequency	Length	Intensity	Assessment type	Pre-test	Post-test
Yu et al. (25)	CHINA (Hong Kong)	five-arm RCT	MCI	MoCA I1: 19.7 (1.9) I2: 18.9 (1.9) I3: 20.1 (1.7) I4: 19.6 (2.5) C: 19.9 (3.5)	I1: 7 I2: 7 I3: 8 I4: 8 C: 7	I1: 63.5 (7.0) I2: 63.5 (5.7) I3: 63.4 (5.2) I4: 63.3 (5.1) C: 63.7 (4.7)	I1: 85.7 I2: 85.7 I3: 87.5 I4: 100 C: 85.7	No	AE walking	I1: Long 150 min I2: Moderate 50 min I3: Long 75 min I4: Short 25 min	I1: Low 1times/week I2: Moderate 3times/weeks I3: Low 1times/week I4: Moderate 3times/weeks	short 12 weeks	I1: Moderate I2: Moderate I3: High I4: High	PSQI	I1: 10.9 (2.1) I2: 9.7 (4.3) I3: 9.9 (3.5) I4: 13.1 (3.5) C: 11.0 (3.5)	I1: 9.1 (2.1) I2: 7.7 (3.4) I3: 6.6 (3.5) I4: 9.3 (3.9) C: 11.6 (4.2)
Bademli et al. (26)	TURKEY	RCT	MCI	MMSE I: 23.27 (2.17) C: 23.42 (1.07)	I: 30 C: 30	I: 72.24 (7.16) C: 70.67 (8.34)	I: 60 C: 56.7	Yes	ME 10-min of warm-up exercises 20-min of rhythmic exercises 10-min of cool down exercises 40-min of walking	Long 80 min	Moderate 3-4times/weeks	Moderate 20 weeks	Moderate	PSQI	I: 9.47 (3.66) C: 8.98 (3.94)	I: 8.33 (3.43) C: 6.76 (2.94)
Chan et al. (27)	CHINA	RCT	MCI	\	I: 25 C: 27	I: 78.4 (7.1) C: 82.2 (6.7)	I: 68 C: 100	Yes	MBE tai chi qigong	Long 60 min	Low 2times/week	Short 2 months	Low	PSQI	\	\
Mu et al. (28)	CHINA	RCT	AD	MMSE I: 18.42 (2.53) C: 18.31 (2.86)	I: 36 C: 36	I: 73.8 (5.3) C: 73.7 (5.1)	I: 58.3 C: 61.1	Yes	AE brisk walking	Short 20-30 min	High 4-6times/weeks	short 12 weeks	low	PSQI	I: 15.47 (4.06) C: 15.53 (3.33)	I: 11.61 (3.60) C: 14.94 (3.53)
Wang et al. (29)	CHINA	two-arm RCT	MCI	MOCA I: 21.65 (2.22) C: 21.41 (2.11)	I: 57 C: 54	I: 68.37 (5.27) C: 68.24 (5.15)	I: 63.2 C: 59.3	No	AE 10-min limbering-up exercises 40-min of upper and lower limbs exercises 10-min relaxation exercises	Long 60 min	Moderate 3times/weeks	Moderate 24 weeks	Moderate	PSQI	I: 9.25 (3.85) C: 8.63 (3.56)	I: 7.77 (2.73) C: 10.22 (2.91)
Wang (30)	CHINA	cluster RCT	MCI	MMSE I:25.03 (2.01) C:24.36 (3.32)	I: 31 C: 30	I: 81.06 (5.17) C: 81.09 (7.44)	I: 78.8 C: 63.6	No	MBE Fitness exercises	Moderate 40 min	Moderate 3times/weeks	Short 12 weeks	Moderate	PSQI	I: 9.12 (3.72) C: 8.61 (4.49)	I: 6.58 (3.36) C: 8.79 (4.39)
Yang et al. (31)	CHINA	RCT	MCI/AD	MMSE I1: 26.64 (2.98) C1: 25.44 (2.35) I2: 17.80 (3.22) C2: 19.78 (3.19)	I1: 9 C1: 11 I2: 9 C2: 10	I1: 70.5 (5.4) C1: 72.0 (8.1) I2: 73.1 (5.2) C2: 75.9 (8.0)	I1: 63.6 C1: 44.4 I2: 80 C2: 77.8	No	MBE sport stacking	Moderate 30 min	Moderate ≥ 5times/weeks	Short 12 weeks	Low	PSQI	I1: 6.36 (3.96) C1: 5.89 (4.31) I2: 6.10 (3.67) C2: 6.73 (2.85)	I1: 5.09 (4.02) C1: 6.33 (4.73) I2: 5.70 (4.20) C2: 8.29 (3.52)
Li et al. (32)	CHINA	RCT	MCI	\	I: 22 C: 19	\	\	No	ME 10-min warm-up exercises 40–50 min of resistance exercise for both upper and lower body10-minute stretching exercises	Long 60 min	Moderate 3times/weeks	Short 12 weeks	Moderate	Actigraphy	TST I: 357.45 (68.38) C: 383.05 (62.56) SE I: 76.03 (8.79) C: 78.76 (12.37) NAT I: 98.50 (43.00) C: 89.63 (60.66)	TST I: 373.64 (79.52) C: 366.37 (73.11) SE I: 84.07 (6.19) C: 75.87 (13.05) NAT I: 56.50 (26.40) C: 104.53 (60.93)
McCurry et al. (33)	United States	RCT	AD	MMSE I: 19.2 (7.7) C: 18.7 (6.9)	I: 32 C: 33	I: 82.2 (8.5) C: 81.2 (8.0)	I: 53 C: 51	Yes	AE Walking	Moderate 30 min	High daily	Moderate 6 months	Low	Actigraphy	TST I: 468.1 (17.7) C: 449.2 (18.8) SE I: 76.0 (2.2) C: 79.9 (2.0) NAT I: 154.0 (16.5) C: 115.5 (12.9)	TST I: 470.6 (21.2) C: 435.6 (20.2) SE I: 77.0 (3.2) C: 78.2 (2.4) NAT I: 146.5 (22.6) C: 122.0 (15.1)

Standard deviation (SD), mild cognitive impairment (MCI), randomized controlled trial (RCT), Alzheimer’s disease (AD), mini-mental state examination (MMSE), intervention (I), control (C), total sleep time (TST), sleep efficiency (SE), nocturnal awakening time (NAT), aerobic exercise (AE), mind–body exercise (MBE), Pittsburgh sleep quality index (PSQI), multi-component exercise (ME), montreal cognitive assessment (MoCA).

### 3.2 Participants

Nine randomized controlled trials comprising 486 participants were meta-analyzed. The mean age of the participants was 73.3 years, and 63.4% were female. MCI was diagnosed according to the National Institute on Aging–Alzheimer’s Association criteria. It was measured using the mini-mental state examination (MMSE) and Montreal Cognitive Assessment (MoCA). Alzheimer’s disease was diagnosed using either the Diagnostic and Statistical Manual of Mental Disorders, fifth edition (DSM-V) or the National Institute of Neurological and Communicative Disorders and Stroke-Alzheimer’s Disease and Related Disorders Association (NINCDS-ADRA). Of the nine studies included in this systematic review and meta-analysis, two reported on AD (28, 33), six enrolled MCI (25–27, 29, 30, 32, 33), and one reported on both AD and MCI (31). Four studies reported sleep problems before exercise interventions (26–28, 33). Study characteristics are detailed in Table 2. Seven of the nine studies used the PSQI as the outcome measurement, while two used Actigraphy.

### 3.3 Exercise characteristics

The present meta-analysis examined the effects of various types of physical exercise on health outcomes. Specifically, four studies employing aerobic exercise (25, 28, 29, 33), three studies on mind–body exercise (27, 30, 31), and two studies utilizing multi-component exercise in conjunction with multiple exercise types (26, 32) were included in the review, without resistance exercise intervention. The exercise time of each intervention ranged from 20–150 min, with frequency ranging from once a week to once a day. The total exercise time ranged from 12 weeks to 6 months, providing a comprehensive evaluation of the long-term effects of physical exercise on health outcomes.

### 3.4 Risk of bias in studies

**Figure 2:**
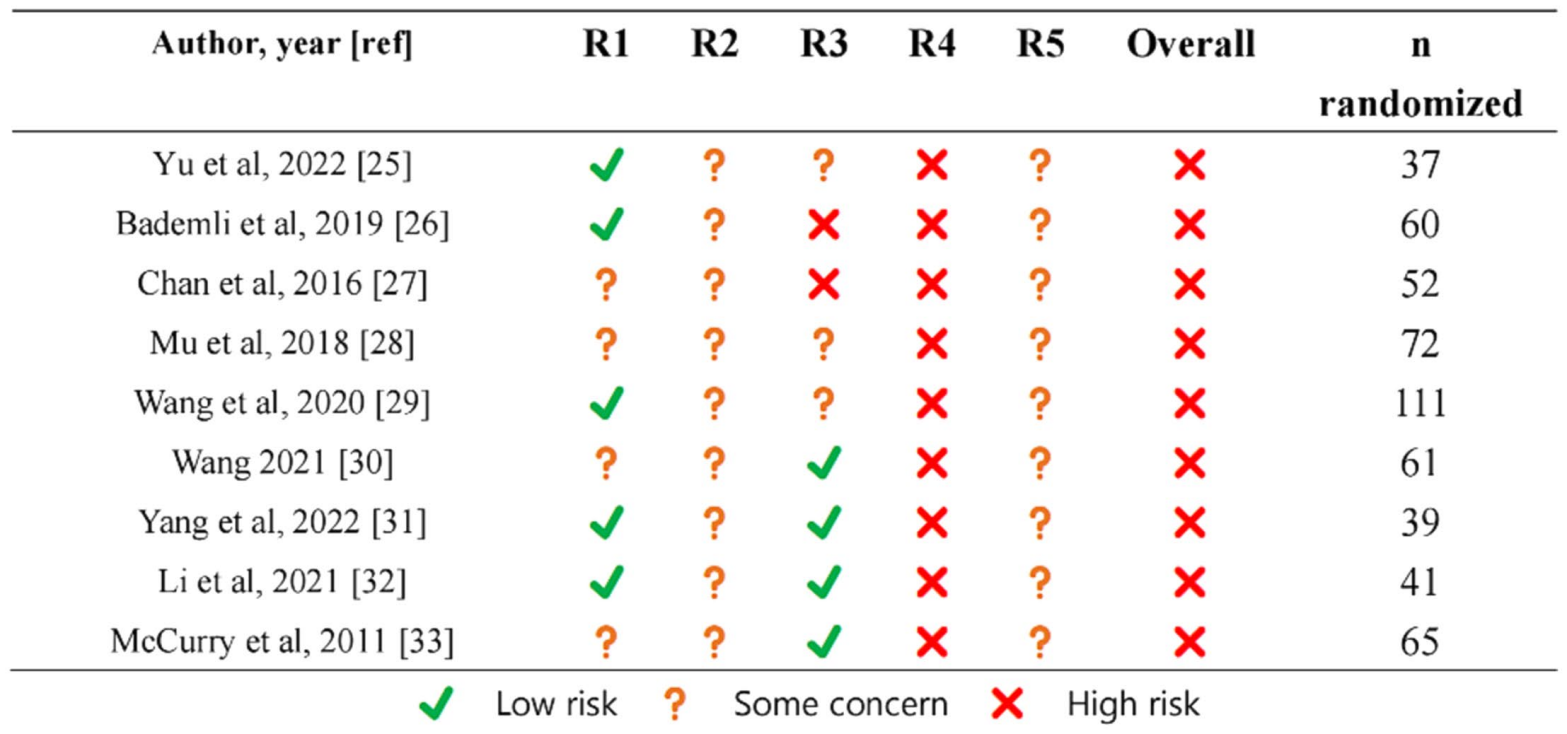
Risk of bias item.

A risk of bias assessment for individual studies is shown in Figure 2.

## 4 Outcomes

### 4.1 Sleep quality

**Figure 3:**
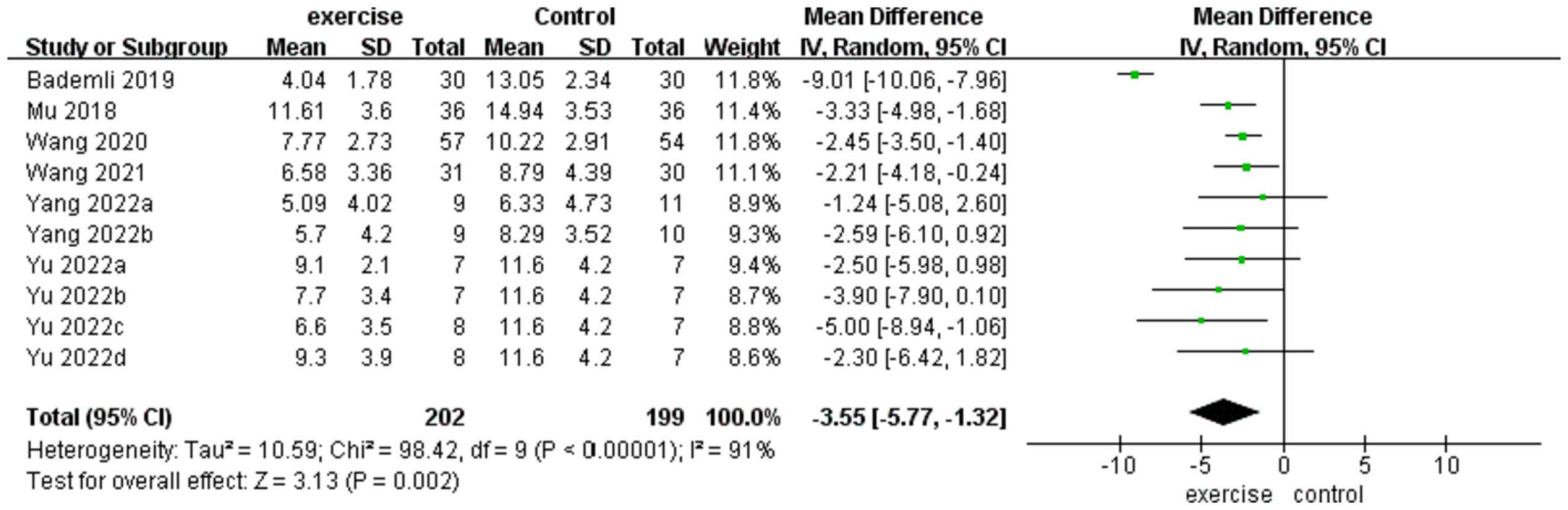
Forest plot of sleep quality.

A meta-analysis comprising six studies (25, 26, 28–31), including a total of 401 participants, was conducted to investigate the impact of physical exercise on sleep quality, as determined by the PSQI. The results revealed a significant improvement in sleep quality [MD = −3.55 (−5.57, −1.32), *Z* = 3.13, *p* = 0.002] among individuals who engaged in physical exercise. However, substantial heterogeneity was observed (*I*^2^ = 91%, P<0.00001), and as a result, we used a random-effects model for analysis, and sensitivity analyses were performed. Notably, after removing the identified outliers (26), sensitivity tests demonstrated significant improvements (*Z* = 7.32, *P* < 0.00001), with I^2^ reduced to 0%. Figure 3 provides a comprehensive forest plot of the effect of physical activity intervention on sleep quality between the intervention and control groups. To further explicate the sources of heterogeneity among studies and identify modifiable factors of physical exercise, subgroup analyses were conducted and presented in detail in Table 3.

**Table 3:** Summary of meta-analysis results of primary and secondary outcomes.

Subgroup	No. of trial (Ref)	Sample Size	MD (95% CI)	Overall effect (value of *p*)	Heterogeneity
PSQI	10 (25, 26, 28–31)	401	−3.55 (−5.77, −1.32)	*Z* = 3.13 (*p* = 0.002)	*I*^2^ = 91% *P*<0.00001
After sensitivity analysis	9 (25, 28–31)	341	−2.68 (−3.39, −1.96)	*Z* = 7.32 (*P* < 0.00001)	*I*^2^ = 0% *p* = 0.92
TST	2 (32, 33)	106	33.77 (23.92, 43.62)	*Z* = 6.72 (*P* < 0.00001)	*I*^2^ = 23% *p* = 0.26
SE	2 (32, 33)	106	2.96 (−6.19, 12.11)	*Z* = 0.63 (*p* = 0.53)	*I*^2^ = 87% *p* = 0.005
NAT	2 (32, 33)	106	−10.36 (−81.38, 60.67)	*Z* = 0.29 (*p* = 0.78)	*I*^2^ = 95% *P* < 0.00001

Reference (Ref), mean deviation (MD), confidence interval (CI), total sleep time (TST), sleep efficiency (SE), nocturnal awakening time (NAT).

### 4.2 Total sleep time and sleep efficiency

A meta-analysis encompassing two RCTs (32, 33), comprising a total of 106 individuals, was conducted to evaluate the impact of physical exercise on total sleep time (TST), sleep efficiency (SE), and nocturnal awakening time (NAT) as assessed by actigraphy. Our findings reveal that physical exercise significantly improves TST [MD = 33.77 (23.92, 43.62), *Z* = 6.72, *P* < 0.00001]. However, no significant differences were observed in SE and NAT. Comprehensive details about these outcomes have been presented in Table 3.

### 4.3 Subgroup analyses

We conducted subgroup analyses on geography, type of cognitive impairment, sleep complaints, duration per exercise, exercise intensity, and exercise frequency of intervention for sleep quality. However, the intervention of ME had a more significant effect on sleep quality than AE and MBE, with statistically significant subgroup differences (*P* < 0.00001).Of note, this analysis revealed no statistically significant differences in the effects of long duration per session (≥60 min) of physical activity on sleep quality [MD = −4.21 (−8.60, 0.18), *Z* = 1.88, *p* = 0.06]. The subgroup analyses of sleep quality are summarized in Table 4.

**Table 4:** Subgroup analyses of meta-analysis results of sleep quality.

Design	Subgroup	No. of trial (Ref)	Sample Size	MD (95% CI)	Overall effect (value of *p*)	Heterogeneity	Subgroup differences (value of *p*)
Geographical regions	China (Mainland)	5 (28–31)	283	−2.56 (−3.33, −1.79)	*Z* = 6.52 (*P* < 0.00001)	*I*^2^ = 0% *p* = 0.83	*p* = 0.22
	Others	5 (25, 26)	118	−4.77 (−8.25, −1.30)	*Z* = 2.69 (*p* = 0.007)	*I*^2^ = 85% *P* < 0.00001	
Type of cognitive dysfunction	MCI	8 (25, 26, 29–31)	310	−3.67 (−6.43, −0.91)	*Z* = 2.61 (*p* = 0.009)	*I*^2^ = 93% *P* < 0.00001	*p* = 0.77
	AD	2 (28, 31)	91	−3.20 (−4.69, −1.71)	*Z* = 4.20 (*P* < 0.0001)	*I*^2^ = 0% *p* = 0.71	
Sleep complaints?	Presence	2 (26, 28)	132	−6.21 (−11.77, −0.64)	*Z* = 2.19 (*p* = 0.03)	*I*^2^ = 97% *P* < 0.00001	*p* = 0.20
	Absence	8 (25, 29–31)	269	−2.52 (−3.32, −1.73)	*Z* = 6.22 (*P*<0.00001)	*I*^2^ = 0% *p* = 0.93	
Type of exercise	AE	6 (25, 28, 29)	241	−2.82 (−3.63, −2.02)	*Z* = 6.87 (*P*<0.00001)	*I*^2^ = 0% *p* = 0.79	*P*<0.00001
	ME	1 (26)	60	−9.01 (−10.06, −7.96)	*Z* = 16.79 (*P*<0.00001)	/	
	MBE	3 (30, 31)	100	−2.12 (−3.69, −0.56)	*Z* = 2.66 (*p* = 0.008)	*I*^2^ = 0% *p* = 0.87	*p* = 0.61
Duration per session	Short	2 (25, 28)	87	−3.19 (−4.72, −1.66)	*Z* = 4.09 (*P* < 0.0001)	*I*^2^ = 0% *p* = 0.65	*P* = 0.61
	Moderate	4 (25, 30, 31)	114	−2.36 (−3.82, −0.90)	*Z* = 3.17 (*P* = 0.002)	*I*^2^ = 0% *p* = 0.82	
	Long	4 (25, 26, 29)	200	−4.21 (−8.60, 0.18)	*Z* = 1.88 (*P* = 0.06)	*I*^2^ = 96% *P* < 0.00001	
Frequency	Low	2 (25)	29	−3.59 (−6.20, −0.98)	*Z* = 2.70 (*P* = 0.007)	*I*^2^ = 0% *p* = 0.35	*p* = 0.98
	Moderate	7 (25, 26, 29–31)	300	−3.51 (−6.52, −0.50)	*Z* = 2.28 (*p* = 0.02)	*I*^2^ = 94% *P* < 0.00001	
	High	1 (28)	72	−3.33 (−4.98, −1.68)	*Z* = 3.96 (*P* < 0.00001)	/	
Intensity	Low	3 (28, 31)	111	−2.94 (−4.33, −1.55)	*Z* = 4.15 (*P*<0.0001)	*I*^2^ = 0% *p* = 0.60	*P* = 0.78
	Moderate	5 (25, 26, 29, 30)	260	−4.09 (−7.69, −0.49)	*Z* = 2.22 (*P* = 0.03)	*I*^2^ = 95% *P* < 0.00001	
	High	2 (25)	30	−3.71 (−6.56, −0.86)	Z = 2.55 (*p* = 0.01)	*I*^2^ = 0% *P* = 0.35	

Reference (Ref), mean deviation (MD), confidence interval (CI), mild cognitive impairment (MCI), Alzheimer’s disease (AD), Aerobic exercise (AE), Mind–body exercise (MBE), Multi-component exercise (ME).

### 4.4 Narrative synthesis

Only one study (28) was excluded from the meta-analysis because of the statistical treatment using the generalized estimating equation model, and the outcome data for the exercise and control groups were pooled. Whereas, we were unable to obtain the data needed for the meta-analysis. We attempted to contact the authors to get it, but unfortunately, we failed. Of note, the study excluded did not demonstrate a significant effect on sleep quality (assessed using the Chinese Pittsburgh Sleep Quality Index, CPSQI score) after 2 months of Tai Chi intervention. However, statistical differences were detected in the CPSQI global score and the sleep duration (*p* = 0.004) and habitual sleep efficiency (*p* = 0.002) subscales at the six-month follow-up. Specifically, exercise participants reported significant improvements in sleep duration (+48 min) and efficiency (+9.1%) during follow-up. The final results were consistent with the current meta-analysis. As with the limitations analysis in Chan’s study (28), the minor improvements in the CPSQI subscale may increase over a more extended training period.

### 4.5 Certainty of evidence

All results were considered moderate except for sleep quality, which was rated low in certainty. Sleep quality was graded as critical because it was the more meaningful indicator of sleep. Total Sleep Time, Sleep Efficiency, and Nocturnal Awakening Time were graded as important (Appendix 1).

## 5 Discussion

To our knowledge, this study is the first systematic review and meta-analysis to provide a comprehensive review of RCT to evaluate the efficacy of various exercise interventions on sleep quality in patients with cognitive impairment. This meta-analysis pooled analysis of the results showed a moderate beneficial effect of physical activity on sleep quality, as indicated by a decrease in PSQI scores. We could observe relative to clinically meaningful improvement in sleep quality. Although there was only one in the included study, ME was considered superior to AE and MBE in subgroup analyses regarding the type of physical activity. The physical activity program chosen in this study (26) consisted of a 10-min warm-up, 20 min of rhythmic exercise, 10 min of cool-down exercise, and 40 min of free walking. This program was designed to be more aligned with the physiological characteristics of individuals aged over 65 years compared to a single form of exercise. It is noteworthy that while no significant differences were observed between the groups in the subgroup analysis for each duration, the analysis demonstrated no statistically significant differences in the effects of long duration per session (≥60 min) of physical activity on sleep quality.

Our meta-analysis on the effect of exercise on sleep quality has considerable heterogeneity, but the source of heterogeneity was clear after sensitivity analysis (26). The effect of exercise on sleep quality was significantly higher in Bademli’s study than in other studies (26). Reviewing the results of the subgroup analysis, we hypothesized that it is likely because of Bademli’s exercise form (Physical Activity Program).

Our analysis revealed a significant beneficial effect of physical exercise on total sleep time but no significant effect on sleep efficiency and nocturnal awakening time. A previous meta-analysis of the effect of exercise on sleep in an average population showed that short-term and regular exercise had statistically significant but minimal effects on total sleep time, sleep efficiency, and nocturnal awakening time (34). However, it is essential to note that our SR-MA was limited by the small sample size, which precluded us from obtaining results on the effect of an exercise intervention on sleep efficiency and nocturnal awakening time. Further research with larger sample sizes and more rigorous study designs is needed to elucidate the potential impact of physical exercise on these objective sleep parameters.

### 5.1 Sleep, exercise, and cognitive impairment

The underlying mechanism of the relationship between exercise and sleep is a complex physiological process involving multiple systems. Consideration of potential mechanisms for the effects of exercise on sleep quality is beyond the scope of this study. Studies suggest that physical exercise improves sleep by regulating the central nervous system, endocrine system, metabolism, mood, and body temperature during sleep (13, 35, 36).

Most previous physical exercise studies on patients with cognitive impairment have focused on cognitive function and functional independence (15, 16), and little attention has been paid to sleep, such as sleep quality and sleep cycle. In recent studies, sleep has been highly correlated with the progression of dementia (37, 38) and the onset of aging (39). With aging, many older adults complain about difficulty initiating or maintaining sleep. (39). For the elderly, sleep, as well as sleep quality, is essential (40, 45).

Studies have shown that sleep promotes the clearance of metabolites in the brain and plays a vital role in the dynamic balance of metabolism in the brain (41). The glymphatic system is a network involved in brain waste removal. This function relies on cerebrospinal fluid (CSF). The glymphatic system is highly correlated with neurodegenerative underlying, including AD (37). Hablitz’s mice experiments showed that circadian rhythms control the cerebrospinal fluid distribution based on the glymphatic system (38). During sleep, CSF is increased into the glymphatic system, facilitating waste clearance (41). A recent study has shown that skeletal muscle can act as an endocrine organ, releasing various muscle cytokines during exercise and exerting anti-inflammatory effects (42). In addition, exercise could prevent acute sleep deprivation-induced inflammatory responses and learning memory deficits and reverse sleep deprivation-induced cognitive decline in acute sleep deprivation-induced inflammatory responses and learning memory deficits. Then, reverse sleep deprivation-induced cognitive decline in cognitive function (43). Sleep also plays a significant role in solidifying memory (44). There is growing evidence for a bidirectional role of sleep disorders in AD pathophysiology (8, 9). Therefore, early intervention of sleep disorders and improving them can not only improve the quality of life of the elderly but also delay the onset of AD.

### 5.2 Limitation

First, the number of included studies and participants was limited. Second, due to the specificity of the sport intervention, the included randomized controlled trials had a high bias. Third, although we have inferred the source of heterogeneity in this study by sensitivity analysis and subgroup analysis, we undeniably had a considerable heterogeneity. Fourth, when number of studies is small (*K* ≤ 10), it is well acknowledged that Egger’s/Peter’s tests or test for funnel plot asymmetry can be underpowered to detect publication bias. However, the funnel plots may be plotted for a visual examination of the small study effect, and no significant publication bias was found (Appendix 2). Fifth, too few studies on objective sleep data from physical activity in patients with cognitive impairment were included for us to accurately derive effects on sleep cycles and rhythms, which would be more clinically meaningful.

## 6 Conclusion

In patients with cognitive impairment, physical exercise may improve sleep quality. ME designed individually based on the physiological characteristics of patients with cognitive impairment was more effective in improving sleep quality. Further large-scale studies with the inclusion of objective sleep outcome will be necessary to evaluate the efficacy of physical exercise on sleep improvement in patients with cognitive impairment.

## Data availability statement

The original contributions presented in the study are included in the article, further inquiries can be directed to the corresponding authors.

## Author contributions

JZ: Writing – original draft. YL: Writing – original draft. QS: Writing – original draft. JS: Writing – original draft. JN: Writing – original draft, Writing – review & editing. TL: Writing – original draft. ZL: Writing – original draft. MW: Writing – review & editing. JT: Writing – review & editing.

## Glossary

GRADE: The Grading of Recommendations Assessment Development and Evaluations
AD: Alzheimer’s disease
SR-MA: Systematic review and meta-analysis
PRISMA: The Preferred Reporting Items for Systematic Reviews and Meta-Analyses
MCI: Mild cognitive impairment
RCT: Randomized controlled trial
AE: Aerobic Exercise
RE: Resistance Exercise
MBE: Mind–Body Exercise
ME: Multi-component Exercise
HRmax: Maximum heart rate
PSQI: The Pittsburgh Sleep Quality Index
MD: Mean differences
SD: Standard deviations
MMSE: The mini-mental state examination
MOCA: Montreal Cognitive Assessment
DSM-V: The Diagnostic and Statistical Manual of Mental Disorders, fifth edition
NINCDS-ADRA: The National Institute of Neurological and Communicative Disorders and Stroke-Alzheimer’s Disease and Related Disorders Association
TST: Total sleep time
SE: Sleep efficiency
NAT: Nocturnal awakening time
CPSQI: The Chinese Pittsburgh Sleep Quality Index
CSF: Cerebrospinal fluid
