# Supplementary material for: Comparative efficacy of various exercise interventions on sleep in patients with cognitive impairment: a systematic review and meta-analysis
Source: Front Neurol. 2024 Feb 1;15:1300459. doi: 10.3389/fneur.2024.1300459 (PMC10867314; doi:10.3389/fneur.2024.1300459)
Supplement: Supplementary file 1 [file Table_1.docx]

| **Comparative efficacy of various exercise interventions on sleep quality in patients with cognitive dysfunction** | | | | | | | | | | | |
| --- | --- | --- | --- | --- | --- | --- | --- | --- | --- | --- | --- |
| **Quality assessment** | | | | | | | **Summary of Findings** | | | | |
| **Participants (studies) Follow up** | **Risk of bias** | **Inconsistency** | **Indirectness** | **Imprecision** | **Publication bias** | **Overall quality of evidence** | **Study event rates (%)** | | **Relative effect** (95% CI) | **Anticipated absolute effects** | |
|  |  |  |  |  |  |  | **With Placebo** | **With Exercise** |  | **Risk with Placebo** | **Risk difference with Exercise** (95% CI) |
| **Sleep Quality** (CRITICAL OUTCOME; Better indicated by higher values) | | | | | | | | | | | |
| 401 (6 studies) | serious^1^ | no serious inconsistency | no serious indirectness | no serious imprecision | reporting bias strongly suspected^2^ | ⊕⊕⊝⊝ **LOW**^1,2^ due to risk of bias, publication bias | 199 | 202 | **-** | - | The mean psqi in the intervention groups was **-3.55** (-5.77 to -1.32) |
| **Total Sleep Time** (IMPORTANT OUTCOME; Better indicated by lower values) | | | | | | | | | | | |
| 106 (2 studies) | serious^1^ | no serious inconsistency | no serious indirectness | no serious imprecision | undetected | ⊕⊕⊕⊝ **MODERATE**^1^ due to risk of bias | 52 | 54 | **-** | - | The mean total sleep time in the intervention groups was **33.77**  (23.92 to 43.62) |
| **Sleep Efficiency** (IMPORTANT OUTCOME; Better indicated by lower values) | | | | | | | | | | | |
| 106 (2 studies) | serious^1^ | no serious inconsistency | no serious indirectness | no serious imprecision | undetected | ⊕⊕⊕⊝ **MODERATE**^1^ due to risk of bias | 52 | 54 | **-** | - | The mean sleep efficiency in the intervention groups was **2.96**  (-6.19 to 12.11) |
| **Nocturnal Awakening Time** (IMPORTANT OUTCOME; Better indicated by lower values) | | | | | | | | | | | |
| 106 (2 studies) | serious^1^ | no serious inconsistency | no serious indirectness | no serious imprecision | undetected | ⊕⊕⊕⊝ **MODERATE**^1^ due to risk of bias | 52 | 54 | **-** | - | The mean nocturnal awakening time in the intervention groups was **-10.36** (-81.38 to 60.67) |

^1^ the specificity of physical exercise interventions
^2^ attrition rates above 20%
