# Supplementary figures and images for: Comparative efficacy of various exercise interventions on sleep in patients with cognitive impairment: a systematic review and meta-analysis
Source: Front Neurol. 2024 Feb 1;15:1300459. doi: 10.3389/fneur.2024.1300459 (PMC10867314; doi:10.3389/fneur.2024.1300459)

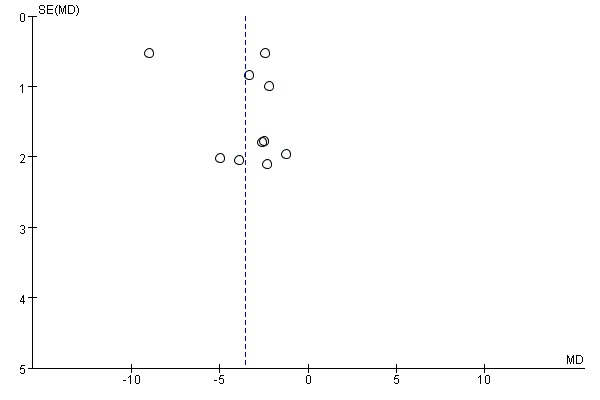

Supplement: Supplementary file 2 [file Image_1.JPEG]
